# The different faces of mental illness stigma: Systematic variation of stereotypes, prejudice and discrimination by type of illness

**DOI:** 10.1111/sjop.12833

**Published:** 2022-05-30

**Authors:** Anke Görzig, Lauren Nicole Ryan

**Affiliations:** ^1^ Psychology and Counselling, School of Human Sciences Old Royal Naval College, University of Greenwich London UK; ^2^ School of Human and Social Sciences University of West London Brentford UK

**Keywords:** stigma, stereotypes, prejudice, mental illness, mental disorders

## Abstract

Mental illness (MI) stigma has been characterized as multi‐dimensional including problems of knowledge (stereotypes), attitudes (prejudice) and behavior (discrimination); however, most research practice is predominantly applying unidimensional behavioral measures such as social distance scales. Moreover, specific types of MIs and different forms of discriminatory behaviors are not being differentiated. The Stereotype Content Model predicts that group stereotypes (warmth, competence) are linked with different forms of discriminatory behaviors (harm or facilitation) via emotional prejudices (pity, envy, contempt). The present study sought to establish how differential stereotypic perceptions of MI subgroups elicit distinct forms of behavioral discrimination via emotional prejudices. A community sample (*N* = 60) was randomly assigned to one of three conditions representing MIs across the warmth‐competence stereotype space. Patterns of self‐completed measures for stereotypes, emotions and behaviors differed significantly between conditions. The association between stereotypes and behaviors were largely mediated by emotions. Systematic patterns of stereotypic perceptions, emotional prejudices and behavioral discrimination are present for individuals with different types of MIs. Hence, generic measures of discrimination, such as social distance scales, may be misleading. Intervention strategies should consider the systematic variation of the factors involved in stigma, differentiating by type of MI and discriminatory behaviors.

## INTRODUCTION

Over a third of the total EU population suffers from mental disorders every year and an increase in sick leave, early retirement and treatment rates due to mental disorders have been reported (Wittchen, Jacobi, Rehm *et al*., 2011). Globally the lifetime prevalence of mental illnesses (MIs) is estimated to be at 29% (Steel, Marnane, Iranpour *et al*., 2014) and one of the main causes of the overall disease burden (Vos, Barber, Bell *et al*., 2015). Individuals with MIs are subjected to stigma and face discrimination (Angermeyer & Dietrich, 2006; Corrigan, 2004; Link, Yang, Phelan, & Collins, 2004; Pescosolido, Martin, Long, Medina, Phelan & Link, 2010). In addition to the effects of the MI itself, negative outcomes are exacerbated for the individual as well as society as a result of stigma. MI stigma has been shown to negatively affect employment, income, resource allocation and healthcare costs (Corrigan, Watson, Warpinski & Gracia, 2004; Sharac, Mccrone, Clement & Thornicroft, 2010). Those with MI are more likely to experience discrimination with regard to housing, employment and see their personal relationships affected as a result of discrimination (Corbière, Zaniboni, Lecomte *et al*., 2011; Corrigan, Larson, Watson, Boyle, & Barr, 2006; Corrigan & Shapiro, 2010; Hamilton, Corker, Weeks *et al*., 2016). MI stigma has further shown to result in self‐stigma, affect well‐being, help‐seeking, treatment compliance and disclosure to others (Brohan, Henderson, Wheat *et al*., 2012; Clement, Schauman, Graham *et al*., 2015; Corrigan, 2004; Evans‐Lacko, Brohan, Mojtabai & Thornicroft, 2012; Livingston & Boyd, 2010). The negative effects of MI stigma are global and persistent over time (Angermeyer, Holzinger & Matschinger, 2010; Pescosolido *et al*., 2010; Thornicroft, Brohan, Rose, Sartorius & Leese, 2009).

Mental illness stigma has been characterized as multi‐dimensional (Angermeyer, Matschinger & Schomerus, 2013; Link *et al*., 2004). It has been described as consisting of problems of knowledge (stereotypes), attitudes (prejudice) and behavior (discrimination). Stereotypes, which are the knowledge or beliefs, whilst prejudicial attitudes include an evaluative (generally negative) element and are the mainly cognitive and affective responses that in turn elicit the behavioral reaction which, in this context, is discrimination (Corrigan, 2004; Thornicroft, Brohan, Kassam & Lewis‐Holmes, 2008). Nonetheless, most research in the area tends to assess MI stigma as unidimensional via social distance scales resembling avoidance behavior assessing discrimination only (Angermeyer & Dietrich, 2006; Angermeyer, Matschinger & Schomerus, 2013; Ross, Morgan, Jorm & Reavley, 2019). These scales assess interpersonal distance in a range of hypothetical scenarios but do not assess stereotypes (knowledge) or prejudice (attitudes). They have been criticized for not assessing actual social decisions and for only roughly approximating experienced behaviors (Corrigan, 2006; Jorm & Oh, 2009; Link *et al*., 2004; Stier & Hinshaw, 2007). Behaviors towards members from discriminated against groups cover a spectrum of different behaviors ranging from more subtle paternalizing behaviors, such as unnecessary or unwanted helping and social exclusion, to overtly hostile behaviors, such as verbal or physical attacks (Fiske, 2012a). Measures of social distance do not cover the range of those differential behaviors.

Moreover, research on MI stigma has been known to examine stigma towards the overarching category of MI as a whole. However, stigma and discriminatory behaviors have been shown to vary by type of MI. This was shown for measures of social distance as well as for stereotypes, emotional reactions and behaviors (Angermeyer, Matthias, Holzinger & Matschinger, 2010; Angermeyer & Dietrich, 2006; Jorm & Oh, 2009; Reavley & Jorm, 2011; Reavley, Morgan & Jorm, 2017). For example, perceptions of threat and responsibility, and associated emotions of fear, anger or pity as well as social distancing behaviors tended to vary between alcohol dependence, dementia, depression, eating disorder and schizophrenia (Angermeyer *et al*., 2010; Angermeyer, Mnich, Daubmann *et al*., 2013; Angermeyer, Matschinger, & Schomerus, 2013; Wood, Birtel, Alsawy Pyle, & Morrison, 2014). However, the underlying mechanisms as well as differential behavioral responses have not been further explored in this line of research.

It has been suggested that MI stigma research should move from the descriptive reporting on social distance to the examination of the links between stereotypes, prejudice and different types of behaviors as well as to further differentiate between the types of MI. Additionally, it has been proposed that stigma research should move away from researching MI stigma as an isolated category and to include comparisons with mentally healthy people in order to assess how MI stigma differs from stigma towards other social groups (Angermeyer & Dietrich, 2006; Corrigan, 2006).

The current research intended to investigate the different dimensions of stigma (stereotypes, emotions and behaviors) whilst establishing in what way different MI are linked with specific types of stigma. Furthering this understanding may open targeted pathways to reducing prejudice and discrimination towards individuals with MI. The Stereotype Content Model (SCM; Fiske, Cuddy, Glick & Xu, 2002) and its later extension the Behaviors from Intergroup Affect and Stereotypes (BIAS) Map (Cuddy, Fiske & Glick, 2007, 2008), widely established theoretical frameworks in social psychology, which offer guidelines for the systematic examination of the different dimensions of stigma (i.e., stereotypes, prejudiced emotions and discriminatory behaviors) will be applied.

### The SCM and the BIAS map

The SCM (Fiske, 2018; Fiske *et al*., 2002) and its later extension the BIAS map (Cuddy *et al*., 2007, 2008) propose a framework that links group stereotypes with specific types of behavioral discrimination that are triggered via emotional responses to the specific group stereotype. Importantly, the SCM explains how different types of discrimination can arise on the basis of a group's stereotype.

The SCM puts forward that when encountering other people or groups, individuals appraise whether their intentions are helpful or harmful (warmth) and whether they are competent or able of enacting them (competence). As a result, two fundamental dimensions have been proposed to systematically explain the stereotypic perceptions of different social groups – warmth (e.g., trustworthiness, friendliness) and competence (e.g., capability, assertiveness). Consequently, the content of the stereotypes about all social groups are represented as a combination of these two dimensions of warmth and competence. Four different quadrants of the warmth‐competence space have been identified: a positive quadrant of high warmth and high competence (HW/HC), a negative quadrant of low warmth and low competence (LW/LC) and two quadrants of mixed or ambivalent stereotypes (i.e., high on one but low on the other dimension), one low in warmth and high in competence (LW/HC) and one high in competence but low in warmth (HC/LW). Social structural variables are thought to causally predict the warmth and competence ratings of groups. People infer (low) warmth from competition (vs. cooperation) and competence from status. Numerous studies have confirmed the warmth‐competence space as well as their socio‐structural antecedents, for example, in representative samples (Cuddy *et al*., 2007), across cultures (Cuddy, Fiske, Kwan *et al*., 2009; Durante, Fiske, Kervyn *et al*., 2013) and subgroups in society (Brambilla, Carnaghi & Ravenna, 2011; Burkley, Durante, Fiske, Burkley & Andrade, 2017; Clausell & Fiske, 2005; Eckes, 2002; Lee & Fiske, 2006).

Each quadrant of the warmth‐competence space is linked with specific emotions. The HW/HC stereotype quadrant generally contains a society's ingroup and allies (e.g., middle class, citizens) and elicits emotions of pride (pride, admiration). The LW/LC quadrant contains societal outcasts (e.g., homeless, immigrants) and is associated with emotions such as contempt and disgust. The two other quadrants, referred to as ambivalent, contain the LW/HC stereotype, which contains successful outsiders, such as entrepreneurs (e.g., rich, professionals) and linked with emotions of envy and jealousy, as well as the HW/LC quadrant, which contains groups, such as the elderly or disabled, and elicits emotions of pity and sympathy (Fiske, 2015, 2018).

The BIAS map has been designed (Cuddy *et al*., 2007) as an extension of the SCM proposing different types of behaviors that are associated with the group stereotypes through the linked prejudicial emotional responses. Four specific types of behaviors are distinguished: active harm (e.g., attacking), passive harm (e.g., ignoring), active facilitation (e.g., helping) and passive facilitation (e.g., cooperating). Warmth is associated with active behaviors and competence with passive behaviors. The behaviors can be harmful or helpful (facilitating); hence, high warmth links with active facilitation (help, protect) and low warmth with active harm (fight, attack). Similarly, high competence is linked with passive facilitation (associate, cooperate) and low competence with passive harm (exclude, demean).

**Fig. 1:**
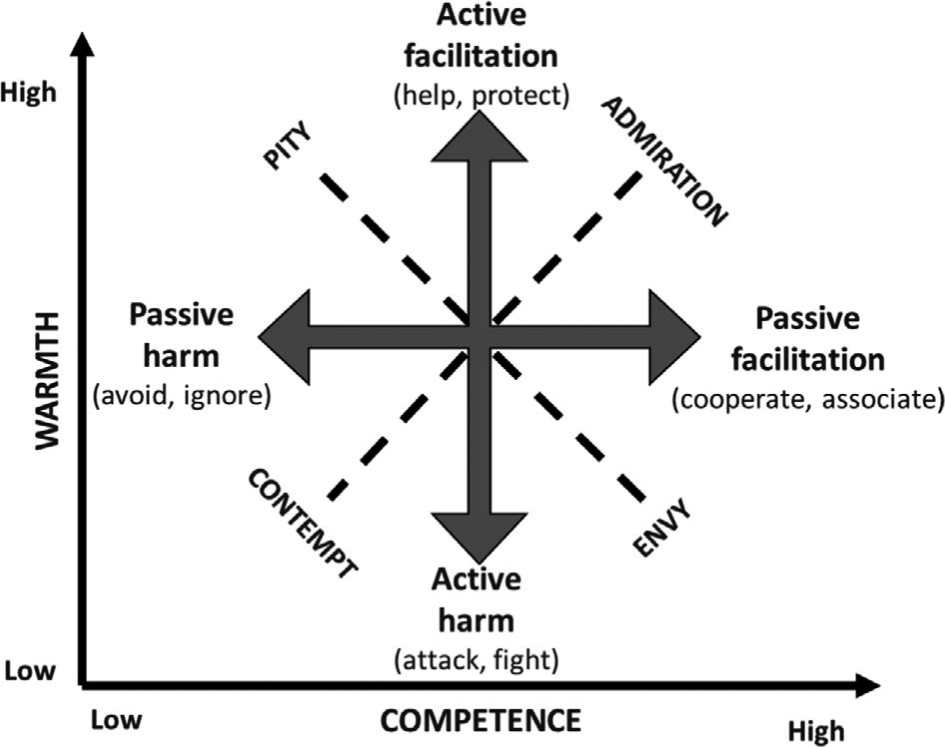
Associations between stereotypes, emotional and behavioral responses according to the BIAS map (Cuddy *et al*., 2007). Stereotypes of warmth and competence are shown on the x‐ and y‐axes. Associated emotions are indicated by dashed lines and displayed in the respective quadrants of the warmth‐competence space. Behaviors are shown by grey arrows alongside the stereotypic dimensions they are associated with. Behaviors are elicited via the emotions displayed in the adjacent quadrants. BIAS, Behaviors from Intergroup Affect and Stereotypes.

Importantly, the SCM proposes that these associations of the stereotypic perceptions of warmth and competence with behaviors are facilitated by or mediated via the emotions outlined above (see Fig. 1 for an illustration). That is, specific emotions are put forward as the mechanisms through which stereotypes elicit specific behaviors. High warmth is associated with active facilitation via admiration (HW/HC) and pity (HW/LC) and low warmth with active harm via envy (LW/HC) and contempt (LW/LC). High competence is associated with passive facilitation via admiration (HW/HC) and envy (LW/HC) and low competence with passive harm via pity (HW/LC) and contempt (LW/LC). When testing for mediational pathways of behavioral tendencies, the BIAS map proposes that each behavioral tendency will be linked to one of the stereotype dimensions via the two associated emotions (e.g., active harm is linked to low warmth as mediated by both, envy and contempt; Cuddy *et al*., 2007, 2008).

Groups whose stereotypic perceptions fall into a specific quadrant of the warmth‐competence space, are proposed to elicit behaviors that are associated with either of the two stereotypic dimension (i.e., the behaviors displayed on either side adjacent to the quadrant, see Fig. 1). For example, a group shown to be stereotypically perceived as low in warmth and low in competence (LW/LC quadrant; e.g., the homeless, drug addicts) will be associated with emotions of contempt or disgust and thereby elicit behavioral responses of active harm (attack, fight; due to low warmth perceptions) as well as passive harm (avoid, ignore; due to low competence perceptions). Another example would be a group shown to be stereotypically perceived as high in warmth but low in competence (HW/LC quadrant; e.g., old or disabled people) will be associated with emotions of pity and thereby elicit behavioral responses of active facilitation (help, protect; due to high warmth perceptions) as well as passive harm (avoid, ignore; due to low competence perceptions).

### SCM and mental illnesses

Research applying the SCM towards individuals from different subgroups of MI is scarce and largely limited to US American samples (Fiske, 2012b; Follmer & Jones, 2017; Sadler, Kaye & Vaughn, 2015; Sadler, Meagor & Kaye, 2012). However, these studies confirmed that different types of MI were indeed perceived differentially in terms of their stereotypes and located at different positions within the warmth‐competence stereotype space. Four cluster solutions emerged that consistently positioned illnesses with psychotic features (e.g., schizophrenia, multiple personality) in the LW/LC cluster whilst those characterized by neuro‐cognitive deficits (e.g., Alzheimer's, mental retardation) were seen similarly low in competence but higher in warmth, falling into a HW/LC cluster. The positioning of MIs associated with internalizing disorders (e.g., anxiety, depression) varied between studies (LW/LC: Follmer & Jones, 2017, Görzig, Bedrošová & Machackova, 2020; HW/LC: Sadler *et al*., 2012, 2015; LW/HC: Fiske, 2012b), most likely due to the prevalence of those MIs among the different samples (i.e., students vs. Mturk). Importantly, those studies did not establish a prototypical ally or ingroup (i.e., HW/HC) cluster and stereotypic perceptions were established relative to the other MIs assessed in the sample. Only one study tested for emotions and behaviors; however, the mediational pathways via the differential set of emotions proposed by the BIAS map (i.e., pity, envy, contempt, admiration) were not established due to differences in procedure and measurement (Sadler *et al*., 2015). None of those studies assessed the socio‐structural components (competition, status) as predictors of stereotypes (warmth, competence).

### Current research

The primary goal of the current research was to explore how differential stereotypic perceptions of MI subgroups may elicit differential emotional prejudices and behavioral discrimination by employing the widely established research paradigm of the SCM and the BIAS map. The current research built on previous research applying the SCM to MI whilst attempting to close the following gaps. A community sample from a different location (UK) was employed to probe transferability of previous findings across samples (Cuddy *et al*., 2009). MI subgroups that have consistently been found to be representative for the primary two cluster locations in the MI stereotype space (i.e., LW/LC and HW/LC) were compared to a control group in order to establish the social structure, stereotypes, emotions and behavioral intentions relative to an ingroup (HW/HC). That is, the subgroups schizophrenia (LW/LC) and Alzheimer's (HW/LC) were selected as representative for their cluster locations and compared with an ingroup (HW/HC) as a control group. Lastly, emotional prejudices and behavioral discrimination were assessed, and the suggested mediational pathways were tested using all four proposed emotional responses in line with the BIAS map in order to fully test for the pathways that may lead to the differential responses to MI subgroups.

### Hypotheses

Firstly, the current research sought to examine whether social structure, stereotypes, emotional prejudice and behavioral discrimination for each of the proposed subgroups would confirm the predictions of the SCM in line with their proposed relative location in the warmth‐competence space. That is, it was hypothesized that compared to the other subgroups:

Hypothesis 1 The schizophrenia subgroup (LW/LC), will be primarily associated with:

(a) social structures of high competition and low status;
(b) stereotypic ratings of low warmth and low competence;
(c) emotional prejudices of contempt; and
(d) behavioral discrimination of active and passive harm.

Hypothesis 2 The Alzheimer's subgroup (HW/LC)will be primarily associated with:

(a) social structures of low competition (cooperation) and low status;
(b) stereotypic ratings of high warmth and low competence;
(c) emotional prejudice of pity; and
(d) behavioral discrimination of active facilitation and passive harm.

Hypothesis 3 The control group (HW/HC)will be primarily associated with:

(a) social structures of low competition (cooperation) and high status;
(b) stereotypic ratings of high warmth and high competence;
(c) emotional prejudice of admiration; and
(d) behavioral tendencies of active and passive facilitation.

Second, it was tested whether the proposed associations of stereotypes with behavioral discrimination via emotional prejudice would hold in line with the BIAS map (Cuddy *et al*., 2007). That is, four further hypotheses were made predicting mediational pathways:

Hypothesis 4 Warmth (high) is hypothesized to predict active facilitation mediated via pity and admiration.

Hypothesis 5 Warmth (low) is hypothesized to predict active harm mediated via contempt and envy.

Hypothesis 6 Competence (high) is hypothesized to predict passive facilitation mediated via envy and admiration.

Hypothesis 7 Competence (low) is hypothesized to predict passive harm mediated via contempt and pity.

## METHODS

### Participants and procedure

Sixty individuals (31 women; age: *M* = 36.48 years, *SD* = 14.99, range 20–75) were recruited via systematic sampling from a retail center in a metropolitan area in the United Kingdom, that is, every 6th person passing by was approached for participation. The majority, 56.7% indicated to be White British, 16.7% Black/Black British, 16.6% Asian/Asian British, 6.7% White Other, and 3.3% mixed heritage. Participants were screened prior to being randomly allocated to one of the three conditions (Alzheimer's, schizophrenia or control; *n* = 20 per condition). Participants were taken into a quieter corner within the retail center and out of earshot from the regular footfall. This was facilitated by the questionnaire itself only taking a few minutes per participant to complete, ensuring that participant confidentiality was recognized. Upon completion of the questionnaire, participants were debriefed and handed information flyers on schizophrenia and Alzheimer's disease which included useful support contacts if needed. After completion, a chocolate bar was given to participants as a reward. Those reporting a personal or family history of mental health illness were excluded from the remainder of the study (*n* < 10). That is, a screening sheet was given, that was presented as a short demographic survey to the willing participants. Those affirming as having a historical background, or any personal experience of MI(es) were thanked for their participation, and given their chocolate bar as a reward upon the screening sheets completion. Ethical approval was obtained by ethics committee of the University of West London and participants provided informed consent prior to participation.

### Materials

Questionnaires were similar across conditions except for the group that scale items referred to, that is, either the two MI subgroups (‘people with schizophrenia,’ ‘people with Alzheimer's disease’) or the control group (‘the average mentally healthy individual’). Questionnaire items from research on SCM and the BIAS Map were used (Cuddy *et al*., 2007) which consisted of 12 two‐item scales using five‐point Likert response scales (1 = not at all; 5 = extremely) whereby the term *group* was replaced with the subgroup specified per condition. Participants were asked to rate these items ‘as viewed by society’ to prevent social desirability bias (cf. Cuddy *et al*., 2007; Fiske *et al*., 2002; for a review of the research paradigm and its applications, see Fiske, 2018). The scales measuring stereotypic traits asked ‘as viewed by society, how [stereotype, e.g., warm] are [group, e.g., people with schizophrenia]’ using the stereotype scales: warmth (warm, friendly; alpha = 0.86), competent (competent, capable; alpha = 0.89). This was followed by the scales measuring social structure asking ‘Again, as viewed by society, how [social structure, e.g., economically successful] have [group] been?’: status (prestigious job, economically successful; alpha = 0.89), competitiveness (special treatment, resources; alpha = 0.72) (cf. Cuddy *et al*., 2007 for the exact wording of each item). Further scales assessed emotional responses asking ‘people tend to feel [emotion, e.g., pity] towards [group, e.g., people with Alzheimer's]’: contempt (contempt, disgust; alpha = 0.85), admiration (admire, proud; alpha = 0.90), pity (pity, sympathy; alpha = 0.79), envy (envy, jealous; alpha = 0.91) and behavioral responses asking ‘people tend to [behavior, e.g., help] [group, e.g., the average mentally healthy individual]’: active facilitation (help, protect; alpha = 0.85), active harm (*f*ight, attack; alpha = 0.91), passive facilitation (cooperate with, associate with; alpha = 0.87), passive harm (exclude, demean; alpha = 0.89). Scales were tested for skewness and kurtosis (Tabachnik & Fidell, 2013). A significant positive skew and kurtosis emerged for envy (*p* < 0.001); hence, the inverse of the scale, multiplied by ‘−1’ to maintain the direction of effects, was used for the remainder of the analyses. A small number of participants (max. *n* = 3) did not complete all items of each scale. That is, one participant did not complete the status scale, two participants did not complete the competition scale and one participant did not complete the active harm scale. All analyses were conducted on complete data only. Given the small number of omissions, procedures for dealing with missing data were not considered.

## ANALYSES AND RESULTS

Our analytic approach followed that of previous work reported in this line of research (i.e., Cuddy *et al*., 2007; Follmer & Jones, 2017; Sadler *et al*., 2015). Although, we were mainly interested in differences and interrelationships between stereotypes, emotions, and behaviors towards people with MI, descriptive statistics and correlations between study variables are included in Table 1, reflecting the original BIAS map research paradigm (Cuddy *et al*., 2007). In line with the BIAS map, status correlated significantly with competence; however, not in line with predictions, but in line with some of previous research on the SCM (Cuddy *et al*., 2009), the correlation between competition and warmth was not significant.

**Table 1:** Pearson's correlations of social structure, stereotypes, emotions and behavioral tendencies

Measure	*M*	*SD*	1.	2.	3.	4.	5.	6.	7.	8.
Social structure										
1. Status	2.31	1.07	‐							
2. Competition	2.91	1.00	−0.32*	‐						
Stereotypes										
3. Competence	2.77	1.21	0.56***	−0.11	‐					
4. Warmth	2.97	1.16	0.25	−0.19	0.16	‐				
Emotions										
5. Contempt	2.83	1.16	−0.25	0.20	−0.38**	−0.38**	‐			
6. Admiration	2.32	1.10	0.60***	−0.27*	0.54***	0.29*	−0.35**	‐		
7. Pity	3.25	1.05	−0.04	−0.10	−0.11	0.36**	−0.23	0.07	‐	
8. Envy	1.66	1.10	0.68***	−0.13	0.57***	0.18	−0.13	0.57***	−0.16	‐
Behavioral tendencies										
9. Active Facilitation	2.93	0.95	0.11	−0.17	0.03	0.57***	−0.41**	0.27*	0.49***	0.02
10. Active Harm	2.70	1.05	0.06	0.23	−0.04	−0.30*	0.52***	0.09*	−0.44**	0.07
11. Passive Facilitation	2.72	1.18	0.80***	−0.35**	0.68***	0.32*	−0.35**	0.69***	0.06	0.65***
12. Passive Harm	3.39	1.10	−0.60***	0.35*	−0.56***	−0.28*	0.43**	−0.37**	−0.08	−0.36**

*Note*: SD = standard deviation.

* *p* < 0.05.

** *p* < 0.01.

*** *p* < 0.001.

### Hypotheses 1–3: subgroup's relative location in the warmth‐competence space

Descriptive statistics for each group on social structure, stereotypes, emotions and behavior along with significant between‐group contrasts are indicated in Table 2. Four separate ANOVAs were conducted to examine Hypotheses 1–3, whether each of the four BIAS map dimensions (social structure, stereotypes, emotional prejudice, and behavioral discrimination) would confirm the predictions of the SCM for each subgroup in line with their proposed relative location in the warmth‐competence space. Condition (subgroups: Alzheimer's, schizophrenia, control) and the BIAS map dimension (social structure: competition, status; stereotypes: warmth, competence; emotions: pity, envy, contempt, admiration; behavioral tendencies: attack, exclude, help, associate) were entered as independent variables using repeated measures for the levels of each of the BIAS map dimensions. Effect coding was used for planned contrasts of single group comparisons. Sample size was determined using a heuristic of 20 participants per cell, and results were not analysed until all responses were collected. Post hoc power analyses were conducted on the interaction effects using G*Power 3.1, indicating all analyses were sufficiently powered (all post hoc power >0.99; Faul, Erdfelder, Lang & Buchner, 2007).

**Table 2:** Social structure, stereotypes, emotions and behaviors by group

	Alzheimer's		Control		Schizophrenia	
	*M*	*(SD)*	*M*	*(SD)*	*M*	*(SD)*
Social structure						
Competition	2.71	(1.08)	2.87	(0.91)	3.13	(1.01)
Status	1.95_a_	(0.81)	3.16_ab_	(1.17)	1.80_b_	(0.66)
Stereotypes						
Warmth	3.85_a_	(0.95)	3.03_a_	(0.90)	2.03_a_	(0.72)
Competence	2.35_a_	(0.97)	3.98_ab_	(0.83)	1.98_b_	(0.83)
Emotions						
Contempt	2.15_a_	(0.88)	2.48_b_	(0.88)	**3.88**_ab_	(0.92)
Pity	**4.10**_ab_	(0.84)	2.98_a_	(0.87)	2.68_b_	(0.89)
Admiration	2.18_a_	(0.98)	**3.05**_ab_	(1.11)	1.73_b_	(0.79)
Envy	1.08_a_	(0.24)	2.80_ab_	(1.24)	1.10_b_	(0.26)
Behaviors						
Active harm	1.95_a_	(0.76)	2.78_a_	(1.01)	**3.42**_a_	(0.82)
Passive harm	**3.13**_a_	(1.00)	2.85_b_	(1.05)	**4.21**_ab_	(0.77)
Active facilitation	**3.70**_a_	(0.66)	**2.80**_a_	(0.91)	2.24_a_	(0.65)
Passive facilitation	2.58_a_	(0.67)	**3.75**_a_	(1.13)	1.76_a_	(0.77)

*Notes*: Means sharing a subscript letter differ significantly between groups (all *p*s < 0.05). Means in bold indicate the emotions and behaviors predicted to be significantly higher for the group indicated in the respective column. SD = standard deviation.

#### Social structure

A 3 (condition) by 2 (social structure) analysis of variance (ANOVA) revealed a significant main effects for condition, *F*(2, 55) = 8.57, *p* = 0.001, *η* ^2^_p_ = 0.24, and structure, *F*(1, 55) = 8.43, *p* = 0.005, η^2^_p_ = 0.13, as well as a significant interaction effect, *F*(2, 55) = 5.27, *p* = 0.008, *η* ^2^_p_ = 0.16. Between‐group contrasts showed no significant differences for competition, all *t*(55)s < 1.3, however, the control group was perceived as significantly higher in status compared to both, the Alzheimer's and the schizophrenia subgroups, *t*(56)s = 4 and 4.69, *p*s < 0.001).

#### Stereotypes

A 3 (condition) by 2 (stereotypes) ANOVA showed a significant main effect for condition, *F*(2, 57) = 29.05, *p* < 0.001, *η* ^2^_p_ = 0.51, but not for stereotype, *F*(1, 57) = 1.75, *p* = 0.19, *η* ^2^_p_ = 0.03, however, there was a significant interaction effect, *F*(2, 57) = 22.07, *p* < 0.001, *η* ^2^_p_ = 0.44. Between‐group contrasts showed that all three groups differed significantly from one another in warmth, *t*(57)s = 2.92–6.46, all *p*s < 0.005) with Alzheimer showing the highest ratings, followed by the control and schizophrenia subgroups. Both, the Alzheimer's and the schizophrenia subgroups were also significantly lower from the control group in competence, *t*(57)s = 6.06 and 7.46, all *p*s < 0.001) whilst they did not differ from one another. Moreover, planned comparisons testing for ambivalent stereotypes (high on one dimension but low on the other) revealed that the Alzheimer's as well as the control subgroups showed a significant difference between warmth and competence, *t*(19)s = 3.50 and 4.74, *p*s < 0.001) whereby the Alzheimer's group was seen as higher in warmth and the control group as higher in competence.

#### Emotions

A 3 (condition) by 4 (emotions) ANOVA revealed significant main effects for condition, *F*(2, 57) = 6.01, *p* = 0.004, *η* ^2^_p_ = 0.18, and emotions, *F*(3, 171) = 40.85, *p* < 0.001, *η* ^2^_p_ = 0.42, as well as a significant interaction effect, *F*(6, 171) = 24.51, *p* < 0.001, *η* ^2^_p_ = 0.46. Between‐group contrasts showed that a higher amount of contempt was expressed towards the schizophrenia group when compared to the control as well as the Alzheimer groups, *t*(57)s = 4.97 and 6.14, *p*s < 0.001, more pity was expressed towards the Alzheimer group when compared to the control as well as the schizophrenia groups, *t*(57)s = 4.11 and 5.21 2.86–7.3, *p*s < 0.001, and lastly, the control group elicited both, more admiration as well as more envy, compared to the Alzheimer and the schizophrenia groups, *t*(57)s = 2.87–7.32, all *p*s < 0.005. Planned comparisons within subgroups revealed that pity was significantly higher for the Alzheimer's subgroup, *t*(19)s = 7.07–16.21, all *p*s < 0.001, and contempt was significantly higher for the schizophrenia subgroup when compared to the other three emotions, *t*(19)s = 4.26–13.03, all *p*s < 0.001. However, no significant differences between emotions were found for the control group, all *t*(19)s < 2.

#### Behavioral tendencies

A 3 (condition) by 4 (behaviors) ANOVA showed no significant effect for condition, *F*(2, 56) = 1.58, *p* = 0.22, *η* ^2^_p_ = 0.05, however, significant effects were revealed for behaviors, *F*(3, 168) = 7.66, *p* < 0.001, *η* ^2^_p_ = 0.12, as well as the interaction effect, *F*(6, 168) = 20.41, *p* < 0.001, *η* ^2^_p_ = 0.42. Between‐group contrasts revealed that all groups differed significantly from one another on active harm with schizophrenia showing the highest ratings followed by the control and Alzheimer's groups, *t*(56)s = 2.32–5.28, all *p*s < 0.05. The schizophrenia group was also significantly higher in passive harm compared to the Alzheimer's as well as the control groups, *t*(56)s = 3.61 and 4.53, *p*s < 0.001. All groups differed significantly from one another on active facilitation with Alzheimer's showing the highest ratings followed by the control and schizophrenia groups, *t*(56)s = 2.21–6.00, all *p*s < 0.05. All groups also differed significantly from one another on passive facilitation with the control group showing the highest ratings followed by the Alzheimer's and schizophrenia groups, *t*(56)s = 2.67–6.85, all *p*s < 0.01. Planned comparisons within subgroups revealed that for the Alzheimer's subgroup the two expected behavioral tendencies were significantly higher than others, insofar as active facilitation was significantly higher than active harm and passive facilitation, whilst passive harm was significantly higher than active harm whilst additionally active facilitation was significantly higher than passive harm, *t*(19)s = 2.21–6.42, all *p*s < 0.05. For the schizophrenia subgroup also the two expected behavioral tendencies, active harm and passive harm, were significantly higher than all other behavioral tendencies whilst passive harm was significantly higher than active harm in addition, *t*(19)s = 2.65–7.42, all *p*s < 0.01. For the control group, one of the expected behavioral tendency, active facilitation, was not significantly higher than any other whilst the other expected behavioral tendency, passive facilitation, showed to be significantly higher than active harm as well as the expected behavior of active facilitation, *t*(19)s = 3.09–4.28, all *p*s < 0.01.

### Hypotheses 4–7: associations of stereotypes with behavioral discrimination via emotional prejudice

In order to test whether the link between stereotypes and behaviors would be mediated via emotions, four separate mediation analyses were conducted, one for each behavioral tendency, analogues to the BIAS map (Cuddy *et al*., 2007). Analyses were conducted using the PROCESS 3.1 macro (Hayes, 2012) in SPSS 21 applying Model 4. All variables were standardized to provide standardized coefficients in the output. The predictor stereotype was entered with the non‐predictor stereotype as a covariate (i.e., warmth was a covariate when competence was a predictor and vice versa). The behavioral tendency was entered as the outcome variable and both emotions predicted to be linked with that behavioral tendency were entered simultaneously as mediators. Direct, indirect and total effects were estimated. The analyses were conducted across all groups. The joint significance approach for mediation was taken to estimate a priori sample size of 58 to detect a medium to large effect (Fritz & MacKinnon, 2007). Post hoc power analyses were conducted on the full regression model including all four predictor variables (i.e., two stereotypes and two emotions) using G*Power 3.1 entering effect sizes (*R* ^2^, see below for each model). Sufficient power was provided for each of the analyses (all post hoc power >0.99; Faul *et al*., 2007).

**Fig. 2:**
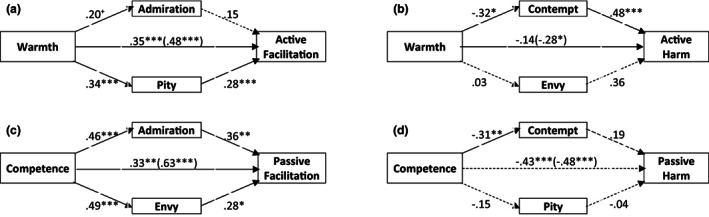
Mediational analyses of the direct effect of stereotypes on behavioural tendencies by emotions. ***p < 0.001, **p < 0.01, *p < 0.05, ^+^ *p* < 0.10. Standardized coefficients are displayed. The coefficient in parentheses represents the direct effect of the stereotype on the behavioral tendency, whereas the adjacent coefficient was observed when emotions were added to the model. The non‐predictor trait was controlled for (i.e., competence was controlled when warmth was the predictor and vice versa). Dashed lines indicate non‐significant effects or non‐significant indirect effects for mediations.

Except for passive harm, one emotion or both significantly mediated the direct effect of the stereotype on the behavioral tendency (see Fig. 2). Warmth was positively associated with active facilitation (*β* = 0.48, *p* < 0.001) which was mediated by pity, total indirect effect = 0.13, 95% CI = [0.03, 0.29], *R* ^2^ = 0.44 (Fig. 2a). Warmth was negatively associated with active harm (*β* = −0.28, *p* < 0.05) which was mediated by contempt, total indirect effect = −0.14,, 95% CI = [−0.31, −0.03], *R* ^2^ = 0.33 (Fig. 2b). Competence was associated with passive facilitation (*β* = 0.63, *p* < 0.001), which was mediated by both envy and admiration, total indirect effect = −0.29, 95% CI = [0.17, 0.43], *R* ^2^ = 0.66 (Fig. 2c). Finally, although a decrease from the total to the direct effect of competence on passive harm (*β* = −0.48, *p* < 0.001) was found, this was neither mediated via contempt nor pity, total indirect effect = −0.05, 95% CI = −0.22, 0.06], *R* ^2^ = 0.38 (Fig. 2d); however, competence showed a negative association with contempt (*β* = −0.31, *p* < 0.01).

## DISCUSSION

The present research explored how differential stereotypic perceptions of MI subgroups elicit differential emotional prejudices and behavioral discrimination. The work built on previous studies applying the SCM (Fiske, 2012b; Follmer & Jones, 2017; Sadler *et al*., 2012, 2015) to subgroups of MI conducted in the US and extended this to the use of a community sample in the UK whilst also using a control group of mentally healthy individuals as a comparison to ratings of MI subgroups. In addition, all mediational pathways using the emotional responses and behavioral tendencies suggested by the theoretical framework (Cuddy *et al*., 2007) were tested in the current study.

### Hypotheses 1–3: subgroup's relative location in the warmth‐competence space

#### Stereotypes and social structure

The lower ratings of both MI subgroups from the control group in competence confirmed expectations and was also seen in the respective differences in status, the antecedent of competence. Expectations were further confirmed for warmth with regard to the MI subgroups of Alzheimer's (high) and schizophrenia (low) as well as for the Alzheimer's group to emerge as an ambivalent stereotype (HW/LC). Against predictions, no group differences in competition emerged. Mirroring the unexpected findings for competition (the antecedent of warmth), the control group was unexpectedly seen as lower on warmth than the Alzheimer's group and emerged as an ambivalent stereotype (LW/HC) due to lower than expected warmth ratings. Given the high prevalence of MI in the population of about 1 in 3 (Wittchen *et al*., 2011), it is likely that most participants, although not identifying with having an MI, had knowledge or were close to someone with MI outside of their immediate family. Hence, the lack of MI in the description of the individual to be rated as the control group, may have led participants to envision an above average individual who may have been perceived as tougher, more competitive, and therefore slightly less warm than the prototypical ingroup.

#### Emotions

Both MI subgroups showed the expected associations with emotional prejudices (pity for Alzheimer's, contempt for schizophrenia) when comparing between groups as well as between emotions. When comparing between groups, the control group showed the expected emotion of admiration; however, unexpectedly envy also emerged. Envy is associated with ambivalent stereotypes in the LW/HC quadrant (Cuddy *et al*., 2007; Fiske *et al*., 2002) which the control group unexpectedly has been shown to be perceived as (see above) and which may explain this additional finding. Moreover, despite showing the highest emotion ratings for admiration and envy between the examined groups, the control group does not appear to have a (statistically significant) main emotion emerging whilst the MI subgroups do. Perhaps, the control group is perceived as more undifferentiated compared to the MI subgroups as it has been described by lacking a trait (i.e., MI) rather than having its own label or identity.

#### Behavioral tendencies

Again, both MI subgroups showed the expected behavioral tendencies to emerge. The schizophrenia group showed the highest ratings for active and passive harm and the Alzheimer's group confirmed the highest ratings for the behavioral tendencies of active facilitation and passive harm. Although, passive harm ratings were slightly lower than expected for the Alzheimer's group, not showing to be significantly higher than passive facilitation within the group or than passive harm for the control group; however, it was still rated significantly lower compared to the schizophrenia group. Similar to the stereotype and emotional ratings, the control group poses an exception insofar as the expected behavior of active facilitation was not rated higher than the behavioral tendencies not expected for this group (i.e., active and passive harm). This finding was substantiated by the control group receiving lower ratings for active facilitation and higher ratings in active harm compared to the Alzheimer's group. It appears that in terms of the warmth‐competence space the lower than expected warmth ratings for the control group may have moved it from the expected HW/HC quadrant towards the LW/HC quadrant, which subsequently enhanced its association with envy and decreased behavioral tendencies for active facilitation whilst increasing those of active harm.

### Hypotheses 4–7: associations of stereotypes with behavioral discrimination via emotional prejudice

#### Mediations

All behavioral tendencies were associated with the stereotypes as predicted and, with the exception of passive harm, were mediated via the respective emotions put forward by the SCM; confirming that emotions were shown to be a stronger predictor of behavioral tendencies than stereotypes. The missing mediation as well as lack of association of contempt and pity with passive harm is puzzling. In particular, as both MI subgroups did show the predicted dominance of the behavioral tendency passive harm, along with the emotional responses (i.e., pity for Alzheimer's, contempt for schizophrenia). Although, contempt was significantly correlated with passive harm, warmth (entered as a control in the mediation) was correlated with both, contempt and passive harm, and may have overridden the effect of contempt in the mediation. This may have been exacerbated by the fact that passive harm ratings for the Alzheimer's group were slightly lower than expected (see above), further explaining the lack of associations with pity, the dominant emotion for this group.

### Limitations and future directions

One of the main limitations evident in the results of the current study is the portrayal of the control group. The control group was intended to serve as a prototypical ingroup but, probably due to its description in terms of lack of a MI instead of representing a societal ingroup in its own right, it was seen to show somewhat lowered ratings in warmth, triggering the emotion of envy and passive but not the anticipated active facilitation, along with general undifferentiated feelings towards this group. Future research in this area should ensure the use of a label which can serve as a true prototypical ingroup.

A further limitation is the small number of MI subgroups used in this study. The current study was a first step in exploring the systematic patterns put forward by the BIAS for MI subgroups. The current selection of MI subgroups was based on their stability across previous studies in terms of their location in the warmth‐competence stereotype space and therefore to represent other MIs in these two main clusters (Fiske, 2012b; Sadler *et al*., 2012, 2015). However, it could thereby not be ascertained whether the established patterns would indeed extend to other MIs found across the stereotype space and whether the established patterns could be generalized to other MIs. Another limitation of the study is the small sample size. Whilst analyses were adequately powered, this did not allow us to assess any further co‐variates that may explain additional within or between group variations. Some of the results may be attributable to the composition of this specific sample. Future research is needed to replicate the current findings across a higher number of MI subgroups with a larger representative sample.

## CONCLUSIONS

Overall, the current study confirmed the systematic patterns of stereotypic perceptions, emotional responses and behavioral tendencies as proposed by the BIAS map for individuals with different types of MI. Moreover, previous research applying the SCM using convenience or student samples in the US has been confirmed by a systematically sampled community sample in another western country (the UK) thereby suggesting that those findings may be more generalizable.

The study's findings suggest that discriminatory behavioral responses systematically differ by type of MI thereby implying that generic measures of discrimination, such as social distance scales, may be misleading as they may not be able to capture the full range of discriminatory behaviors (Corrigan, 2006; Jorm & Oh, 2009; Link *et al*., 2004; Stier & Hinshaw, 2007). Moreover, it was confirmed that prejudiced emotional responses are the primary predictors of such behaviors towards individuals with MI (Cuddy *et al*., 2007; Thornicroft *et al*., 2008) and that, similar to the behaviors, emotional responses vary systematically by type of MI in line with the associated stereotypes. Hence, when considering discriminatory behaviors towards individuals with MI, emotional responses as well as stereotypes towards different MI subgroups may need to be taken into account. Previous recommendations of MI researchers to take account of all factors representing stigma, such as knowledge (stereotypes), attitudes (prejudice) and behavior (discrimination) (Corrigan, 2004; Thornicroft *et al*., 2008), as well as to differentiate responses by type of MI (Angermeyer & Dietrich, 2006), have been corroborated by the current study.

Emotional prejudices can take on the forms of contempt, pity or envy and accordingly discriminatory behaviors can vary from overt attacks to social exclusion or paternalizing behaviors. All of which can negatively impact on the well‐being of individuals with MI as well as their economic and social burden on society as a whole (Corbière *et al*., 2011; Corrigan *et al*., 2006, 2004; Corrigan & Shapiro, 2010; Hamilton *et al*., 2016; Sharac *et al*., 2010). This has important implications for interventions. Many national and international health policies include the elimination of stigma and anti‐stigma campaigns have been on the rise (Corrigan, Morris, Michaels, Rafacz, & Rüsch, 2012; Mehta *et al*., 2015). Taking into account the systematic variation of the factors involved in stigma and differentiating by type of MI needs to be an integral part of these campaigns if discrimination against individuals with MI is to be addressed at the core. The change of social‐structural factors and stereotypes as the causal antecedents of emotions that trigger those discriminatory behaviors (Caprariello, Cuddy, & Fiske, 2009) appears to be a promising avenue for improvements.

This article builds on the second author's Undergraduate dissertation at the University of West London (Ryan, 2015).

Ethical approval was obtained by the Ethics Committee at the University of West London and participants provided informed consent prior to participation.

## Data Availability

The data that support the findings of this study are available from the corresponding author upon reasonable request.

## References

[sjop12833-bib-0001] Angermeyer, M.C. & Dietrich, S. (2006). Public beliefs about and attitudes towards people with mental illness: A review of population studies. Acta Psychiatrica Scandinavica, 113, 163–179. 16466402 10.1111/j.1600-0447.2005.00699.x

[sjop12833-bib-0002] Angermeyer, M.C. , Mnich, E. , Daubmann, A. , Herich, L. , Wegscheider, K. , Kofahl, C. *et al*. (2013). Biogenetic explanations and public acceptance of people with eating disorders. Social Psychiatry and Psychiatric Epidemiology, 48, 1667–1673. 23296547 10.1007/s00127-012-0648-9

[sjop12833-bib-0003] Angermeyer, M.C. , Holzinger, A. & Matschinger, H. (2010). Emotional reactions to people with mental illness. Epidemiologia e Psichiatria Sociale, 19, 26–32. 20486421 10.1017/s1121189x00001573

[sjop12833-bib-0004] Angermeyer, M.C. , Matschinger, H. & Schomerus, G. (2013). Attitudes towards psychiatric treatment and people with mental illness: Changes over two decades. British Journal of Psychiatry, 203, 146–151. 10.1192/bjp.bp.112.122978 23787060

[sjop12833-bib-0005] Brambilla, M. , Carnaghi, A. & Ravenna, M. (2011). Status and cooperation shape lesbian stereotypes. Social Psychology, 42, 101–110.

[sjop12833-bib-0006] Brohan, E. , Henderson, C. , Wheat, K. , Malcolm, E. , Clement, S. , Barley, E.A. *et al*. (2012). Systematic review of beliefs, behaviours and influencing factors associated with disclosure of a mental health problem in the workplace. BMC Psychiatry, 12, 11. 22339944 10.1186/1471-244X-12-11 PMC3298486

[sjop12833-bib-0007] Burkley, E. , Durante, F. , Fiske, S.T. , Burkley, M. & Andrade, A. (2017). Structure and content of Native American stereotypic subgroups: Not just (ig)noble. Cultural Diversity and Ethnic Minority Psychology, 23, 209–219. 27429064 10.1037/cdp0000100

[sjop12833-bib-0008] Caprariello, P.A. , Cuddy, A.J.C. & Fiske, S.T. (2009). Social structure shapes cultural stereotypes and emotions: A causal test of the stereotype content model. Group Processes and Intergroup Relations, 12, 147–155. 10.1177/1368430208101053 PMC3839230 24285928

[sjop12833-bib-0009] Clausell, E. & Fiske, S.T. (2005). When do subgroup parts add up to the stereotypic whole? Mixed stereotype content for gay male subgroups explains overall ratings. Social Cognition, 23, 161–181.

[sjop12833-bib-0010] Clement, S. , Schauman, O. , Graham, T. , Maggioni, F. , Evans‐Lacko, S. , Bezborodovs, N. *et al*. (2015). What is the impact of mental health‐related stigma on help‐seeking? A systematic review of quantitative and qualitative studies. Psychological Medicine, 45, 11–27. 24569086 10.1017/S0033291714000129

[sjop12833-bib-0011] Corbière, M. , Zaniboni, S. , Lecomte, T. , Bond, G. , Gilles, P.‐Y. , Lesage, A. *et al*. (2011). Job acquisition for people with severe mental illness enrolled in supported employment programs: A theoretically grounded empirical study. Journal of Occupational Rehabilitation, 21, 342–354. 21656251 10.1007/s10926-011-9315-3

[sjop12833-bib-0012] Corrigan, P. (2004). How stigma interferes with mental health care. American Psychologist, 59, 614–625. 15491256 10.1037/0003-066X.59.7.614

[sjop12833-bib-0013] Corrigan, P.W. (2006). Mental health stigma as social attribution: Implications for research methods and attitude change. Clinical Psychology: Science and Practice, 7, 48–67.

[sjop12833-bib-0014] Corrigan, P.W. , Larson, J.E. , Watson, A.C. , Boyle, M. & Barr, L. (2006). Solutions to discrimination in work and housing identified by people with mental illness. The Journal of Nervous and Mental Disease, 194, 716–718. 16971826 10.1097/01.nmd.0000235782.18977.de

[sjop12833-bib-0015] Corrigan, P.W. , Morris, S.B. , Michaels, P.J. , Rafacz, J.D. & Rüsch, N. (2012). Challenging the public stigma of mental illness: A meta‐analysis of outcome studies. Psychiatric Services, 63, 963–973. 23032675 10.1176/appi.ps.201100529

[sjop12833-bib-0016] Corrigan, P.W. & Shapiro, J.R. (2010). Measuring the impact of programs that challenge the public stigma of mental illness. Clinical Psychology Review, 30, 907–922. 20674114 10.1016/j.cpr.2010.06.004 PMC2952670

[sjop12833-bib-0017] Corrigan, P.W. , Watson, A.C. , Warpinski, A.C. & Gracia, G. (2004). Stigmatizing attitudes about mental illness and allocation of resources to mental health services. Community Mental Health Journal, 40, 297–307. 15453083 10.1023/b:comh.0000035226.19939.76

[sjop12833-bib-0018] Cuddy, A.J.C. , Fiske, S.T. & Glick, P. (2007). The BIAS map: Behaviors from intergroup affect and stereotypes. Journal of Personality and Social Psychology, 92, 631–648. 17469949 10.1037/0022-3514.92.4.631

[sjop12833-bib-0019] Cuddy, A.J.C. , Fiske, S.T. & Glick, P. (2008). Warmth and competence as universal dimensions of social perception: The stereotype content model and the BIAS map. Advances in Experimental Social Psychology, 40, 61–149.

[sjop12833-bib-0020] Cuddy, A.J.C. , Fiske, S.T. , Kwan, V.S.Y. , Glick, P. , Demoulin, S. , Leyens, J.P. *et al*. (2009). Stereotype content model across cultures: Towards universal similarities and some differences. British Journal of Social Psychology, 48, 1–33. 19178758 10.1348/014466608X314935 PMC3912751

[sjop12833-bib-0021] Durante, F. , Fiske, S.T. , Kervyn, N. , Cuddy, A.J.C. , Akande, A.D. , Adetoun, B.E. *et al*. (2013). Nations' income inequality predicts ambivalence in stereotype content: How societies mind the gap. British Journal of Social Psychology, 52, 1–24. 23039178 10.1111/bjso.12005 PMC3855559

[sjop12833-bib-0022] Eckes, T. (2002). Paternalistic and envious gender stereotypes: Testing predictions from the stereotype content model. Sex Roles, 47, 99–114.

[sjop12833-bib-0023] Evans‐Lacko, S. , Brohan, E. , Mojtabai, R. & Thornicroft, G. (2012). Association between public views of mental illness and self‐stigma among individuals with mental illness in 14 European countries. Psychological Medicine, 42, 1741–1752. 22085422 10.1017/S0033291711002558

[sjop12833-bib-0024] Faul, F. , Erdfelder, E. , Lang, A.G. & Buchner, A. (2007). G* power 3: A flexible statistical power analysis program for the social, behavioral, and biomedical sciences. Behavior Research Methods, 39, 175–191. 17695343 10.3758/bf03193146

[sjop12833-bib-0025] Fiske, S.T. (2012a). Managing ambivalent prejudices. American Psychologist, 639, 33–48. 10.1177/0002716211418444 PMC3792573 24115779

[sjop12833-bib-0026] Fiske, S.T. (2012b). Warmth and competence: Stereotype content issues for clinicians and researchers. Canadian Psychology, 53, 14–20. 24155504 10.1037/a0026054 PMC3801417

[sjop12833-bib-0027] Fiske, S.T. (2015). Intergroup biases: A focus on stereotype content. Current Opinion in Behavioral Sciences, 3, 45–50. 27453920 10.1016/j.cobeha.2015.01.010 PMC4955357

[sjop12833-bib-0028] Fiske, S.T. (2018). Stereotype content: Warmth and competence endure. Current Directions in Psychological Science, 27, 67–73. 29755213 10.1177/0963721417738825 PMC5945217

[sjop12833-bib-0029] Fiske, S.T. , Cuddy, A.J.C. , Glick, P. & Xu, J. (2002). A model of (often mixed) stereotype content: Competence and warmth respectively follow from perceived status and competition. Journal of Personality and Social Psychology, 82, 878–902. 12051578

[sjop12833-bib-0030] Follmer, K.B. & Jones, K.S. (2017). Stereotype content and social distancing from employees with mental illness: The moderating roles of gender and social dominance orientation. Journal of Applied Social Psychology, 47, 492–504.

[sjop12833-bib-0031] Fritz, M.S. & MacKinnon, D.P. (2007). Required sample size to detect the mediated effect. Psychological Science, 18, 233–239. 17444920 10.1111/j.1467-9280.2007.01882.x PMC2843527

[sjop12833-bib-0032] Görzig, A. , Bedrošová, M. & Machackova, H. (2020). Do mental illness stereotypes predict bystander behaviour in cyber‐bullying? An application of the stereotype content model. International Journal of Developmental Science, 13, 83–95.

[sjop12833-bib-0033] Hamilton, S. , Corker, E. , Weeks, C. , Williams, P. , Henderson, C. , Pinfold, V. *et al*. (2016). Factors associated with experienced discrimination among people using mental health services in England. Journal of Mental Health, 25, 350–358. 26854361 10.3109/09638237.2016.1139068

[sjop12833-bib-0034] Hayes, A. F. (2012). PROCESS: A versatile computational tool for observed variable mediation, moderation, and conditional process modeling. Retrieved May 2022 from http://www.afhayes.com/public/process2012.pdf

[sjop12833-bib-0035] Jorm, A.F. & Oh, E. (2009). Desire for social distance from people with mental disorders. Australian & New Zealand Journal of Psychiatry, 43, 183–200. 19221907 10.1080/00048670802653349

[sjop12833-bib-0036] Lee, T.L. & Fiske, S.T. (2006). Not an outgroup, not yet an ingroup: Immigrants in the stereotype content model. International Journal of Intercultural Relations, 30, 751–768.

[sjop12833-bib-0037] Link, B.G. , Yang, L.H. , Phelan, J.C. & Collins, P.Y. (2004). Measuring mental illness stigma. Schizophrenia Bulletin, 30(3), 511–541. 15631243 10.1093/oxfordjournals.schbul.a007098

[sjop12833-bib-0038] Livingston, J.D. & Boyd, J.E. (2010). Correlates and consequences of internalized stigma for people living with mental illness: A systematic review and meta‐analysis. Social Science & Medicine, 71(12), 2150–2161. 21051128 10.1016/j.socscimed.2010.09.030

[sjop12833-bib-0039] Mehta, N. , Clement, S. , Marcus, E. , Stona, A.‐C. , Bezborodovs, N. , Evans‐Lacko, S. *et al*. (2015). Evidence for effective interventions to reduce mental health‐related stigma and discrimination in the medium and long term: Systematic review. British Journal of Psychiatry, 207, 377–384. 10.1192/bjp.bp.114.151944 PMC4629070 26527664

[sjop12833-bib-0040] Pescosolido, B.A. , Martin, J.K. , Long, J.S. , Medina, T.R. , Phelan, J.C. & Link, B.G. (2010). ‘A disease like any other’? A decade of change in public reactions to schizophrenia, depression, and alcohol dependence. American Journal of Psychiatry, 167, 1321–1330. 20843872 10.1176/appi.ajp.2010.09121743 PMC4429867

[sjop12833-bib-0041] Reavley, N.J. & Jorm, A.F. (2011). Stigmatizing attitudes towards people with mental disorders: Findings from an Australian national survey of mental health literacy and stigma. Australian & New Zealand Journal of Psychiatry, 45, 1086–1093. 22023236 10.3109/00048674.2011.621061

[sjop12833-bib-0042] Reavley, N.J. , Morgan, A.J. & Jorm, A.F. (2017). Predictors of experiences of discrimination and positive treatment in people with mental health problems: Findings from an Australian national survey. Social Psychiatry and Psychiatric Epidemiology, 52, 269–277. 27803976 10.1007/s00127-016-1301-9

[sjop12833-bib-0043] Ross, A.M. , Morgan, A.J. , Jorm, A.F. & Reavley, N.J. (2019). A systematic review of the impact of media reports of severe mental illness on stigma and discrimination, and interventions that aim to mitigate any adverse impact. Social Psychiatry and Psychiatric Epidemiology, *54*, 11–31. 30349962 10.1007/s00127-018-1608-9

[sjop12833-bib-0044] Ryan, L. N. (2015). Distinct mental illnesses evoke systematic patterns of stereotype content and emotional prejudice among the lay public: an empirical investigation using the Stereotype Content Model and the BIAS map for Alzheimer and schizophrenia subgroups. Thesis, University of West London, UK

[sjop12833-bib-0045] Sadler, M.S. , Kaye, K.E. & Vaughn, A.A. (2015). Competence and warmth stereotypes prompt mental illness stigma through emoti.ons. Journal of Applied Social Psychology, 45, 602–612.

[sjop12833-bib-0046] Sadler, M.S. , Meagor, E.L. & Kaye, K.E. (2012). Stereotypes of mental disorders differ in competence and warmth. Social Science and Medicine, 74, 915–922. 22321391 10.1016/j.socscimed.2011.12.019

[sjop12833-bib-0047] Sharac, J. , Mccrone, P. , Clement, S. & Thornicroft, G. (2010). The economic impact of mental health stigma and discrimination: A systematic review. Epidemiologia e Psichiatria Sociale, 9, 223–232. 10.1017/s1121189x00001159 21261218

[sjop12833-bib-0048] Steel, Z. , Marnane, C. , Iranpour, C. , Chey, T. , Jackson, J.W. , Patel, V. *et al*. (2014). The global prevalence of common mental disorders: A systematic review and meta‐analysis 1980–2013. International Journal of Epidemiology, 43, 476–493. 24648481 10.1093/ije/dyu038 PMC3997379

[sjop12833-bib-0049] Stier, A. & Hinshaw, S.P. (2007). Explicit and implicit stigma against individuals with mental illness. Australian Psychologist, 42, 106–117.

[sjop12833-bib-0050] Tabachnik, B. G. , & Fidell, L. S. (2013). Using multivariate statistics: Pearson new international edition. Using multivariate statistics. Harlow: Pearson Education Limited. 10.1037/022267

[sjop12833-bib-0051] Thornicroft, G. , Brohan, E. , Kassam, A. & Lewis‐Holmes, E. (2008). Reducing stigma and discrimination: Candidate interventions. International Journal of Mental Health Systems, 13, 3. 10.1186/1752-4458-2-3. PMC2365928 18405393

[sjop12833-bib-0052] Thornicroft, G. , Brohan, E. , Rose, D. , Sartorius, N. & Leese, M. (2009). Global pattern of experienced and anticipated discrimination against people with schizophrenia: A cross‐sectional survey. The Lancet, 373, 408–415. 10.1016/S0140-6736(08)61817-6 19162314

[sjop12833-bib-0053] Vos, T. , Barber, R.M. , Bell, B. , Bertozzi‐Villa, A. , Biryukov, S. , Bolliger, I. *et al*. (2015). Global, regional, and national incidence, prevalence, and years lived with disability for 301 acute and chronic diseases and injuries in 188 countries, 1990–2013: A systematic analysis for the global burden of disease study 2013. The Lancet, 386, 743–800. 10.1016/S0140-6736(15)60692-4 PMC4561509 26063472

[sjop12833-bib-0054] Wittchen, H.U. , Jacobi, F. , Rehm, J. , Gustavsson, A. , Svensson, M. , Jönsson, B. *et al*. (2011). The size and burden of mental disorders and other disorders of the brain in Europe 2010. European Neuropsychopharmacology, 21, 655–679. 21896369 10.1016/j.euroneuro.2011.07.018

[sjop12833-bib-0055] Wood, L. , Birtel, M. , Alsawy, S. , Pyle, M. & Morrison, A. (2014). Public perceptions of stigma towards people with schizophrenia, depression, and anxiety. Psychiatry Research, 220, 604–608. 10.1016/j.psychres.2014.07.012. 25064387

